# Developing nurse match: A selection tool for evoking and scoring an applicant's nursing values and attributes

**DOI:** 10.1002/nop2.183

**Published:** 2018-08-07

**Authors:** Colin McNeill, Allen Erskine, Roger Ellis, Marian Traynor

**Affiliations:** ^1^ Identity Exploration Ltd. Belfast Northern Ireland UK; ^2^ University of Ulster Newtownabbey UK; ^3^ School of Nursing and Midwifery Queen's University Belfast Belfast UK

**Keywords:** nursing applicants, personal nursing values, professional identity in nursing, professional nursing values, qualitative methodology, scoring personal values, screening for selection, UCAS personal statement, values‐based recruitment

## Abstract

**Aim:**

To develop an instrument (Nurse Match: NM) for assessing a candidate's nursing values, their meaning, relative importance and emotional significance. Candidate's values to be scored against professionally preferred nursing values effectively and efficiently.

**Design:**

A case study‐based qualitative process with quantified output. Perception of self and others in relevant contexts using bi‐polar value dimensions.

**Methods:**

Respondents (*N *=* *63) were first year nursing students completed the instrument and a feedback questionnaire. Data were analysed and scored by ipseus software using algorithm defined parameters. Statistical analysis: Minitab 17.

**Results:**

The instrument

- discriminated effectively and efficiently between year one nurses in terms of the professional quality of their inherent nursing values and attributes;
- created suitability scores (S^TOT^ scores) for candidate screening purposes;
- suitability scores closely approximated normal distributions;
- was valid and reliable: robust in quantitative and qualitative terms;
- was administered, scored and interpreted in a standard manner;
- was easy to understand and complete and well received by participants.

The NM instrument offers a standardized, effective, user friendly, screening process for values and attributes. Development work with a group of actual applicants is required. NM is complementary to other modes of assessment.

## 1 INTRODUCTION

The purpose of this research is to develop Nurse Match (NM), a self‐report instrument for values‐based recruitment: assessing a candidate's nursing values, their meaning, relative importance and emotional significance. Development is in the context of concern that standards in nursing may be falling and a need to identify candidates with attributes suggesting suitability for the work and cultural fit.

The long‐term goal is an appraisal process with universal relevance, assessing fit of a candidate from any background with localized culturally and socially appropriate nursing values.

### 1.1 Background

Values‐Based Recruitment (VBR) is an important programme of work in the National Health Service in the UK. It was devised after a mandate from government to Health Education England (HEE) to “deliver high quality, effective, compassionate care: developing the right people with the right skills and the right values”; (Department of Health, 2013a).

It is an approach which “attracts and selects students, trainees or employees on the basis that their individual values and behaviours align with the values of the NHS Constitution”, (Department of Health, 2013b). It is about “enhancing existing processes to ensure that the NHS recruits the right workforce not only with the right skills and in the right numbers, but with the right values to support effective team working and excellent patient care and experience” (HEE, 2016).

There has been an increased focus on the values agenda across the NHS in the UK, in part due to the Francis Report, (Francis, 2013), which highlighted the vital role of the workforce in providing high quality and safe health care. The report repeatedly emphasized the importance of staff values and behaviours for the level of care and patient experience; see on benchmarks (Department of Health, 2010a) and best practice (Department of Health, 2010b) and on professional standards of practice and behaviour, (Nursing and Midwifery Council, 2015) Nursing and Midwifery Council.

The Department of Health, Social Services and Public Safety (DHSSPS) Education Strategy Group (ESG) identified a need for streamlining the application and selection processes for Higher Education Institutions (HEIs) during 2011. The Northern Ireland Practice and Education Council for Nursing and Midwifery (NIPEC) was commissioned by the ESG to undertake a project to develop a strategy which would optimize efficiency of application and selection processes to identify individuals who display attributes that are valued.

Phase Two of that project focussed on the “attributes which are valued to realize future potential in a career in nursing”. The NIPEC report to ESG, (NIPEC, 2014), considered that it had “added to the growing evidence in relation to the attributes that could be used in selecting students”. The values and attributes used in the project are set out in a NIPEC report (Northern Ireland Practice and Education Council for Nursing and Midwifery (NIPEC), 2014): see below under *Measures* for the values and attributes as applied in this pilot.

Evidence that some personal attributes of student nurses such as empathy and moral orientation were unchanged after 3 years, (Pitt, Powis, Levett‐Jones, & Hunter, 2014), points up the importance of incorporating the assessment of these qualities into selection and recruitment of nursing students.

However, despite the obvious importance of right and wrong conduct, a recent comprehensive review of literature on professional ethics in nursing concluded that “professional ethics has not been studied much in nursing science. Greater knowledge of professional ethics is needed to understand and support nurses’ moral decision‐making and to respond to the challenges of current changes in health care and society”, (Kangasniemi, Pakkanen, & Korhonen, 2015).

A paper by (Ellis, Griffiths, & Hogard, 2015) located the foundations for work on a new value‐based instrument in theoretical and empirical terms, reviewed available instruments and recommended Weinreich's Identity Structure Analysis (ISA), applied using Ipseus software: see also (Passmore, Ellis, & Hogard, 2014) and (Hogard, 2014). The Ipseus software is universal in its application in that it facilitates the design of bespoke instruments for the practical investigation of nursing identity and preferred nursing values in a wide range of social and cultural settings.

Here, the focus is on its use in a value‐based appraisal of applicants to schools of nursing in the United Kingdom. While values may modulate across cultures and between schools of nursing, the process of assessment and selection used here can be applied using any set of culturally relevant, professionally preferred, values.

The Ellis paper described the nursing values used in the new instrument, called Nurse Match (NM), their derivation in the literature and the nursing profession, how the instrument offers an in‐depth analysis of the respondent's position regarding key nursing values and how initial results using an early version of the instrument demonstrated its power to identify and distinguish value orientations of individuals.

The nursing values being used in the Ellis version of NM were representative of the NIPEC attributes and were mapped to the NIPEC themes as set out in Table 2 below. The in‐depth identity analysis offered by NM goes quite a way beyond what could be managed for a large cohort of respondents being more suited to a case study or developmental work with an individual.

## 2 THE STUDY

### 2.1 Aims

The primary objective of this work was therefore the development and assessment of a simple effective and efficient measure, easily understood and managed: a way of systematically appraising a respondent's personal nursing values against a set of values preferred by the profession, in this case six NIPEC “themed attributes” — “Person centredness” (PC), “Accountability” (ACC), “Trust” (T), “Integrity” (I), “Commitment to personal development” (CPD) and “Teamwork” (TW).

It was also important to confirm that the instrument appeared valid to respondents as well as easy to understand and complete and so we sought feedback.

The immediate aim of this research was improvement and further development of the NM instrument as a tool and a process for screening applicants to nursing from a variety of social and cultural backgrounds for their personal values and appraise those against established local standards in terms of nursing values and attitudes. It is believed that this approach has the flexibility necessary to operate effectively in the context of population movements and an international dynamic that is modulating extant local value systems.

A secondary objective was to explore the relationship between cohort scores on the pilot instrument and on two other measures of nursing potential; the personal statement and the MMI interview process.

### 2.2 Design

**Figure 1:**
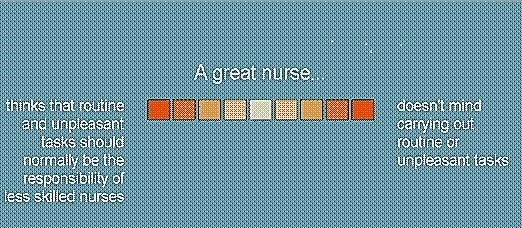
An example of a nursing value presented using a semantic differential scale

A case‐study approach to screening for values that required respondents to appraise themselves and relevant others using an instrument (NM) designed to explore personal use of nursing values and attributes (see Figure 1). Ipseus software was used to record responses and report the outcome and three theoretical concepts (ISA) used to score the data.

#### 2.2.1 Measures

Two measures were used.

**Figure B1:**
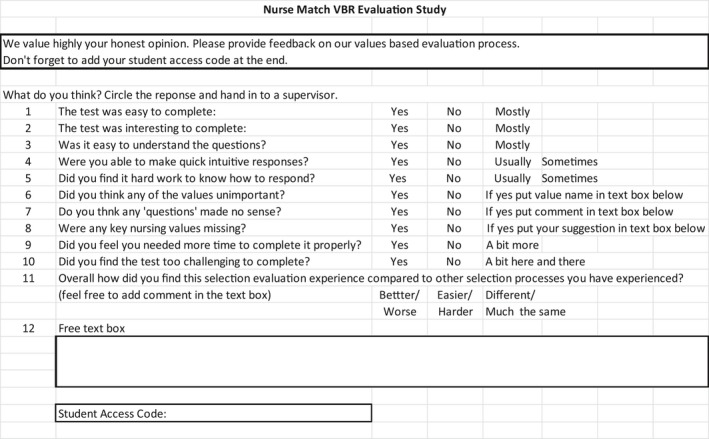
The questionnaire used by participants when providing feedback on their experience of using the Nurse Match instrument

1 Nurse Match (NM) an ISA instrument, custom designed and built using the Ipseus software framework was used to apply:
  - twenty (20) bi‐polar constructs representing nursing attributes and values (see Figure 1) to
  - thirteen (13) entities from personal, home and work domains (Table 1) and
2 a Feedback Questionnaire (Appendix B: Figure B1).

**Table 1:** The entities used in the nurse match instrument

	Relevant entities (including aspects of self)
01	Ideal self
02	Self at work
03	Self at home
04	Self under pressure
05	Me 2 years ago
06	Me in 5 years’ time
07	The person I most dislike
08	A model nurse
09	A ward sister
10	A typical patient
11	A bad nurse
12	My best friend
13	My parents

The value constructs used in the NM instrument were derived from a literature search, trials with experienced and well‐respected nurses and life experience, see Figure 1 for an example. They were aligned with NIPEC attributes and values.

Each attribute or value was presented as a “dimension” connecting two contrasting points of view (a construct). Respondents used a nine‐point, semantic differential scale with centre zero. A response scored from 1‐4 on the point of view it represented, 1 being a weak response close to zero and 4 a strong one. One pole of each construct consisted of a preferred professional attribute or value. Respondents indicated the attribute they personally preferred when appraising “aspirational self”. Their personal preference may or may not have been a professional preference. The centre zero was used by the respondent if they could not decide between polar values.

The entities are aspects of self and people from the workplace and home context. Respondents were asked to appraise aspects of self and other people in terms of the attributes or values they perceive them to have or hold e.g., “At work I … am prepared to challenge someone more senior if I feel it is in the interests of the patient/… would not challenge someone more senior in any circumstances”.

The set of aspirations, self's value preferences, were used as a benchmark in a scoring process that compared them with the set of professionally preferred values.

Data output was presented by the software (Ipseus) in an automated report that used the entity/construct matrix of scores (+4 to −4) on responses appraising self and others to calculate scores on ISA parameters. Scores on two selected ISA parameters (value stability — sp.; and emotional significance — es.) together with ideal self's choice of pole were used to calculate a score (S) for each of the twenty NM nursing values.

Each of the six value themes (Person Centredness PC, Accountability ACC, Trust T, Integrity I, Commitment to Personal Development CPD, Teamwork TW) is composed of a set of nursing attributes and values (see Table 2 below). These themes were recommended by the School of Nursing having proved their worth in Phase Two of the ESG project (Northern Ireland Practice and Education Council for Nursing and Midwifery (NIPEC), 2014).

**Table 2:** Constructs making up the six value themes

Nursing values by construct	Person centredness	Accountability	Trust	Integrity	Commitment to personal development	Teamwork
(C)	(P)	(ACC)	(T)	(I)	(CPD)	(TW)
C1	X	X	X			
C2	X	X	X			
C3		X				X
C4						X
C5	X	X				
C6					X	
C7	X					
C8		X		X		
C9	X	X				
C10		X		X		
C11		X			X	
C12		X	X		X	
C13		X	X	X		
C14	X					X
C15	X					X
C16		X	X	X		X
C17		X	X	X		X
C18		X				X
C19		X			X	X
C20	X					X
Use of C by value theme	8	14	6	5	4	9

The score for each value theme (S^TOT^) is the sum of the S scores on the constituent values. The mean of the six S^TOT^ scores is the individual's score on NM for assessment purposes. See Appendix A: Table A2 for an example of the calculation.

Note re Nurse Match (NM): An exposition of the Nurse Match measure including the full wording of the set of bi‐polar constructs used and the scoring process is available from the principal author on request.

The second measure was a feedback questionnaire (see Appendix B: Figure B1). It was completed by all respondents immediately following completion of the instrument. A free text box was available.

### 2.3 Sample

#### 2.3.1 Respondents

The respondents (*N* = 63) were first year students at the Queen's University Belfast School of Nursing and Midwifery nearing the end of the final semester of the year.

These students were a convenience sample from the September 2014 cohort of successful applicants. They had been selected for interview using personal statements and appraised at structured selection interviews. Many of the cohort (*N* = 110), had volunteered to participate in a pilot of an MMI value‐based assessment process based on the same nursing values used in the NM instrument (Traynor et al., 2017). Our volunteers (*N* = 63) were a subset of the MMI volunteers. They were therefore well‐positioned to provide feedback on the MMI and NM Values‐Based Recruitment (VBR) selection processes.

The NM study was held after the MMI pilot procedure that took place on the 23rd March 2015. Those respondents (*N* = 110) who had taken part in the MMI study were asked by School of Nursing staff if they were willing to participate and they were offered the inducement of a free lunch of sandwiches and coffee and participation in a draw for retail vouchers of £100 and £200 respectively. The NM research was granted approval by the School of Nursing and Midwifery Ethics Committee. Sixty‐three students agreed to participate.

### 2.4 Data collection

The September 2014 cohort entering the School of Nursing and Midwifery were all given Student Unique Identifier (SUI) numbers which were used to identify their personal statement scores, their MMI scores and their scores on the NM instrument. And, subsequently, their scores on end of year modules.

On 5 May 2015, 63 first year students completed the NM pilot instrument in a group setting (a computer laboratory). The Ipseus software was downloaded and the NM instrument was completed by all 63 respondents each of whom were seated at a desk with their own terminal, well‐spaced out in a computer room at QUB. A presentation was delivered to brief all respondents on the procedure to be followed. Respondents were requested not to consult on responses. Immediately after completion of the instrument, each respondent completed a feedback questionnaire (see Appendix B; Figure B1). A free text box was available.

### 2.5 Ethical considerations

Ethics committee approval was obtained from the university.

### 2.6 Data analysis

Initial data analysis was carried out automatically by Ipseus software using algorithms defining two ISA concepts (“structural pressure” on a construct and “emotional significance” of a construct) to calculate a score on each concept for each value construct. That data, downloaded to Excel software, were used with the chosen pole of each construct to calculate a score for each respondent on each of the six value themes and an overall score; the chosen pole may or may not have been the professionally preferred pole. Subsequent statistical analysis used Minitab 17. The output is an S^TOT^ score for each respondent for each of the six nursing value themes together with a mean S^TOT^ score for the set of value themes: see Table 3 below.

### 2.7 Validity and reliability

#### 2.7.1 Theoretical basis

The epistemological position we adopt is essentially constructionist. That is a person develops a unique sense of self and perspective by way of personal experience of self‐in‐the‐world (see ISA meta‐theory: Weinreich & Saunderson, 2003). Sense of identity and construal of the world emerges from the activity of real world somatic and neuropsychological processes that develop and modulate self's values and beliefs in response to life experience.

#### 2.7.2 Methodology

We use ISA in an idiographic case‐study approach to each applicant. Data are gathered using a structured self‐report instrument. This approach offers a holistic description of self‐identity in a constrained context that is then open to interpretation and analysis. The complexity of outcome and subjective judgement involved in interpretation of interrelated data mean that, practically, findings are often focussed on and limited to narrower aspects of self‐identity. In this research, for practical reasons related to aim, the report is limited to appraisal of the applicant's value and belief system with outcome limited to a graphic illustrating scores on nursing themes and an overall score estimating the match or fit of self's values and beliefs with those required of a nurse. The result is in fact a snapshot in time of an aspect of a personal value and belief system that is relevant to nursing. If you like, we are exploring self‐identity but for now only looking at self's construal of self and life in the context of nursing. `

#### 2.7.3 Qualitative rigour

The criteria we adopted to address validity and reliability, provide qualitative rigour, are based accordingly on this philosophical position and methodology and expressed here in terms of the literature (Anney, 2014; Huntley et al., 2017; Madill, Jordan, & Shirley, 2000; O'Brien, Harris, Beckman, Reed, & Cook, 2014; Pidgeon & Henwood, 1995; Tai & Ajjawi, 2016; Tobin & Begley, 2004). We used a selection of SRQR criteria:

1. *Credibility*: the credibility of our chosen values was established by way of an extended process of exploration and selection (see below under robustness). The score on a value construct is an estimate of its meaning to the respondent and its emotional significance for them and is “true” to the extent that the respondent's response is natural.
2. *Fitness:* the designed, quantitative, in face, content and construct validity of the instrument has been re‐affirmed by feedback on its use (immediately post hoc instrument completion) and this is closely bound up with the fitness for purpose of the qualitative elements. The degree of “stability” of each qualitative element (value construct) is estimated using a clearly defined scoring process, so that value construct and personal score on it combine to create valid meaning couched in ISA terms.
3. *Robustness:* the ISA systematic procedure is inherently robust, but a great deal of time and effort was expended to ensure that the values used in the study were credible, dependable, confirmable and transferable (Anney, 2014; Houghton, Casey, Shaw, & Murphy, 2013). This was done during an extended developmental period; exploring the literature on values, consulting with experienced nurses and with several leaders in the profession, using personal experience of the NHS, creating a list of 102 value constructs, reducing that to twenty by a process of content analysis, reducing those twenty to six themes related to attribute themes being used in the NHS, having the professionals complete pilot instruments and provide feedback and seeking confirmation through feedback from the group who sat this pilot instrument — which generally confirmed that the values used in the study had face and content validity and were indeed “credible, dependable, confirmable and transferable”.
4. *Reliability*: the instrument provides a reliable “snapshot” of values at time of response; reliability of the “snapshot” over time remains to be fully appraised. We are realistic about the stability of a unique idiographic measure such as this considering all the personal and social factors in play over time. We are conscious of research in social psychology and neuroscience on the role of values and evaluation in thinking and decision‐making, (House, 2016; Kahneman & Egan, 2011). The intuitive process that operates automatically with little apparent effort and can respond quickly detecting simple relationships and the more considered process that can follow rules, compare objects on several attributes and make deliberate choices. We expect that the more stable and simple values will have continuity over time in the more settled identity while the less stable and more complex values will be relatively easily changed or adjusted by mood and circumstance.
5. *Integrity*: genuineness of approach to completion of the instrument is the key factor in integrity. We advise respondents to respond promptly and intuitively which helps to avoid “overthinking”. Lack of integrity of response is detectable by way of the software report and is usually due to lack of willingness to engage with the process. Feedback indicated that the instrument was easy to complete and understand (98% and 95% of respondents).
6. *Representativeness:* this is an idiographic exploration so not generalizable from individual to the group but scoring in ISA is standardized to make individual's scores comparable with others using the same process; see Weinreich (Weinreich & Saunderson, 2003) at pp. 78–9, 104–5 and 110 on “standardization” and “internal standardization”.
7. *Coherence:* the findings on significance of value constructs are the consequence of a “synthesis” by algorithm assessing (a) the importance and (b) the emotional significance of each value in the appraised of self and others across several contexts: workplace, home, social life, past_self, future_self, self_under_ pressure.
8. *Transparency:* it is clear where the data came from and how results are reported.

## 3 THE FINDINGS

### 3.1 Values‐based appraisal: data output

A subset of the data is presented in Table 3 as an illustration of initial output before rank ordering. The output of all cohort scores prepared for screening is set out, rank ordered, in Appendix A: Table A1. The outcome is an S^TOT^ score for each nursing value theme and an overall, mean, S^TOT^ score. Use of the output is a matter for the screening agent.

**Table 3:** Section of NM values‐based results table before rank ordering

Theme/Student	PC	ACC	T	I	CPD	TW	MEAN
SUI0001	85.61	61.48	50.76	54.48	51.59	60.96	60.81
SUI0003	76.81	68.98	70.90	57.47	72.13	58.75	67.51
SUI0004	32.34	13.80	7.07	−12.90	32.71	18.34	15.22
SUI0005	46.42	35.19	24.25	38.34	43.14	33.70	36.84
SUI0006	54.58	43.23	36.03	45.08	35.65	39.85	42.40
SUI0007	82.32	62.52	65.57	40.21	89.73	68.26	68.10

**Figure 2:**
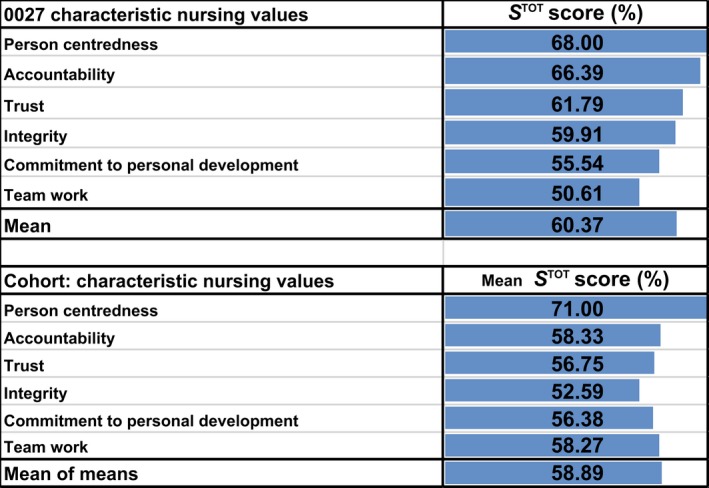
Example of S^TOT^ Scores (SUI0027) compared with Cohort Scores

The results for individuals can be easily compared with scores for the cohort. Either as a simple rank ordering as in Appendix A: Table A1 or they can be presented in a more informative manner as individual cf. cohort scores — See Figure 2 above.

#### 3.1.1 Statistical properties of the data

See Table 4 for a set of basic descriptive statistics: note similar standard deviations (SD) of nursing values. There is a moderate to strong positive and statistically significant relationship between respondent S^TOT^ scores on value themes i.e., one theme variable tends to increase as the other increases (see Table 5).

**Table 4:** Simple descriptive statistics for the S^TOT^ scores on the themed values

Themed nursing value	Min	Max	Mean	SD
Person centredness (PC)	32	98	73	14
Accountability (AC)	14	91	60	15
Trust (T)	7	98	58	19
Integrity (I)	−13	93	54	19
Commitment to personal development (CPD)	9	94	58	19
Teamwork (TW)	18	90	60	14
Mean	11	94	61	17

**Table 5:** Pearson correlation between respondent's S^TOT^ scores on NM value themes

Moderate to strong correlations: significance; *p*‐value = 0.001 for all							
	PC	ACC	T	I	CPD	TW	Mean^a^
PC		0.85	0.695	0.716	0.515	0.85	0.852
ACC	0.85		0.886	0.868	0.758	0.866	0.984
T	0.695	0.886		0.762	0.79	0.724	0.928
I	0.716	0.868	0.762		0.458	0.836	0.872
CPD	0.515	0.758	0.79	0.458		0.513	0.779
TW	0.85	0.866	0.724	0.836	0.513		0.887
Mean^a^	0.852	0.984	0.928	0.872	0.779	0.887	

a the mean here is the respondent's overall score on the six themes.

**Figure 3:**
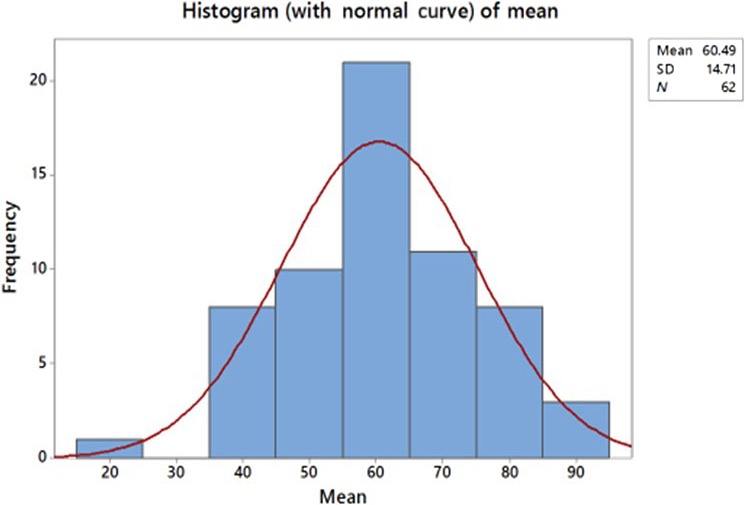
Distribution of overall scores determining rank ordering of respondents

S^TOT^ scores on each value theme and the overall (mean) scores on value themes have a distribution that approximates to normal. Because of constraints on space, only the histogram of the overall (mean) scores is offered as evidence see Figure 3 (*N* = 62 one participant withdrawn). Rank ordering of respondents is determined by this overall score.

#### 3.1.2 Secondary correlation study

This was to explore the linear relationships between measures of nursing competence: personal statements, MMI selection interviews and end year module scores. Work on correlation though not completed (limited to NM cf. MMI) indicates several weak and significant but unhelpful correlations, two of which are negative: see Table 6. Scores on “Accountability”, assessed using MMI, correlate significantly and positively, but weakly (*r* = 0.25), with scores on “Person Centredness” assessed using NM and correlate significantly but weakly and negatively (*r* = −0.28 and −0.27 respectively) with “Integrity” and “Teamwork” assessed using NM. The weak negative correlation of “Accountability” (MMI) with “Accountability” (NM) is not significant (*p* = 0.06). The evidence is that there is little or no linear relationship between “equivalent” themed values.

**Table 6:** NM and MMI scores on the themed values; correlation and significance

Pearson r: 5% significance level								
		PC	% scores on MMI values			CPD	TW	GLOBAL^a^
			ACC	T	I			
S^TOT^ scores on NM values	PC_1	0.17	0.25	−0.09	−0.08	0.11	−0.12	−0.11
	*p* value	0.19	0.05	0.50	0.52	0.42	0.38	0.41
	ACC_1	−0.11	−0.24	−0.09	−0.13	0.15	−0.12	−0.04
	*p* value	0.41	0.06	0.49	0.33	0.25	0.38	0.74
	T_1	0.01	−0.19	0.02	−0.02	0.12	−0.11	0.07
	*p* value	0.94	0.14	0.87	0.87	0.37	0.42	0.60
	I_1	−0.08	−0.28	−0.14	−0.11	0.11	−0.04	−0.05
	*p* value	0.54	0.03	0.30	0.42	0.41	0.78	0.73
	CPD_1	0.08	−0.07	0.06	−0.05	0.17	−0.07	0.09
	*p* value	0.53	0.58	0.67	0.71	0.19	0.61	0.48
	TW_1	−0.08	−0.27	−0.1	−0.07	0.11	−0.1	−0.04
	*p* value	0.56	0.04	0.46	0.57	0.41	0.44	0.74
	MEAN_1^b^	−0.05	−0.24	−0.06	−0.09	0.15	−0.1	−0.01
	*p* value	0.68	0.06	0.67	0.52	0.27	0.45	0.97

a The Global score is the score attributed to an applicant by the observer on completion of all six MMI stations assessing values, expressed as a % of the maximum score possible.

b The Mean_1 score is the NM equivalent of the MMI Global Score i.e., the mean of all six scores on NM as a % of maximum score. One correlation test is between applicant's ‘global’ MMI and ‘mean’ NM scores.

##### Comment

This lack of any systematic and clear relationships typifies what is being found with MMI selection interviews, personal statement and end year module scores (evidence on the two latter modes is indicative only and not reported here). NM and MMI do not appear to be measuring the same thing although they purport to be doing so. For example, scores on “Person Centredness” (PC) assessed subjectively in mini‐interview by an observer do not correlate well with scores on “Person Centredness” (PC) assessed “objectively” in an ISA self‐report case study.

### 3.2 Feedback

The responses to the questionnaire on the experience of completing the pilot instrument were collated and the data on responses to the questions analysed (see feedback questionnaire at Appendix B: Figure B1). See comment below and Table 7 for a summary of the findings and comparison with the MMI feedback.

**Table 7:** MMI and NM: Feedback from students compared

	MMI	NM	
Student comment (*N* = 110)	%	%	Student comment (*N* = 63)
A positive experience	86	98	Easy to complete
A fair assessment tool	79	95	Easy/mostly easy to understand
Tested their suitability for profession	74	94	No key nursing values missing
Could show understanding better than interview	71	90	Interesting to complete
Better way to select than current style of interview	58	90	Not too challenging to complete
Unsure about this	31	84	Responses easy intuitive
		83	Issues raised were important
Assessors comment		81	All questions asked made sense
Wide range of attributes	92	81	Not hard work sometimes testing
Appropriate way of Assessing	81	10	Had a little bit of difficulty here and there
		8	Felt they needed more time to complete
			Free text
			Different: puzzling questions: obvious answers to a nurse
			Better or worse than MMI was conflicted (20% for MMI)

**Figure B2:**
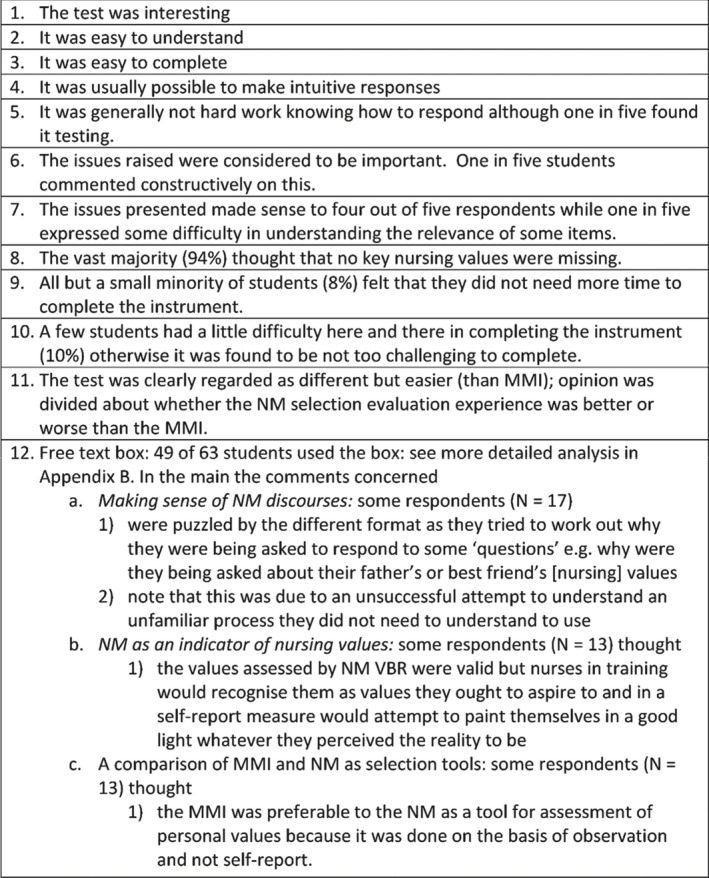
Summary of the findings about participant's experience of using the instrument and their opinion of it.

Text from the “free text box” was reviewed and the findings summarized in Appendix B: Figure B2 (at 12). Summary of feedback: the NM instrument was seen by most respondents to:

1. have face value and
2. identify most important nursing values,
3. be interesting,
4. be easy to understand and complete and
5. be a “different experience”.

Free text comment concerned

1. the format, which some (26%) found puzzling
2. the values: some (20%) thought they were valid but nurses in training would recognize those they ought to aspire to and paint themselves in a good light
3. the MMI process, as a tool for assessment of personal values; 20% preferred it because assessment was based on observation of “real situations” and not self‐report.

Note: the puzzle over format was due to an unsuccessful attempt to understand a process the respondents did not need to understand to use the test. Also, NM and MMI measure the same value themes from two different perspectives, the personal (NM) and an observers (MMI) and so would be complementary.

## 4 DISCUSSION

The primary aim of the work was to continue development of Nurse Match by piloting the instrument with a cohort of first year nursing students scoring the resulting value profiles and obtaining feedback on user experience.

### 4.1 Strengths

The choice of NM values (nursing attributes) and value themes is well aligned with recently researched attributes and value themes.

Presentation and use of the instrument worked well, most respondent's being fully engaged and responding appropriately. The scoring process for comparing respondent profiles on nursing values discriminated effectively between nursing students with no tied scores. Descriptive statistics produced a normal distribution of scores overall and for each NIPEC theme value, with appropriate ranges, means and variances.

The instrument had and was seen by respondents to have face and content validity identifying important nursing values, was interesting, easy to understand and complete and was said to be a different experience. As a self‐report measure, it was complementary to other modes of assessment.

### 4.2 Limitations

Just asking people directly about themselves and what they think of others can offer revealing, fascinating and rich data. By their very nature internal identity states including values cannot be observed directly. Valid and reliable self‐reports rely on sound motivation, openness, honesty and astute self‐awareness which is difficult to ensure. Self‐report knowledge of a person's mind set, world view and perception is always valuable information. However, particularly in respect of those whose identity development has yet to mature and stabilize, it's validity and reliability may be a judgement best made by others observing and discussing behaviour in the workplace.

Suitability in terms of nursing values and attributes can only be part of the process of assessment. There is clearly a need for complementarity in appraisal of nurses and candidates for both developmental and recruitment purposes.

A secondary aim was to explore the relationship between cohort scores on the pilot NM instrument and two other measures of nursing competence; personal statement scores and the MMI selection process. Initial indications were of low or no linear relationship, positive or negative, between Nurse Match appraisal of values and the other measures of nursing competence.

It seems that different modes of assessment have been of some practical use in recruitment and selection, but each says something different about the characteristics of the candidate and their potential as a nurse. A multifaceted approach that compiles a set of tests and measures each having a defined purpose with validity and predictive value does seem sensible. The quest being to find the best and most suitable cluster of tests.

## 5 CONCLUSION

The Nurse Match instrument piloted here effectively discriminated between student nurses and rank ordered them by appraising and scoring their personal nursing values against a set of professionally preferred values. The test was found by student nurses to be easy to use and valid in construct terms. Scores showed a normal distribution.

The next stage in development of the instrument will be a larger scale replication study with a cohort of new applicants to nursing. Applicants will be screened and scored on the suitability of their personal values to a career in nursing and the outcomes, robustness and efficacy of Nurse Match compared with the UCAS Personal Statement process. Grant funding has been received for this work and is underway with a 2018 cohort of applicants.

The longer term aim is to establish the credentials of the Nurse Match process universally and make it available globally to schools of nursing as a simple, low resource, value‐based screening process for use in candidate selection.

Despite the general difficulties with effective selection measures, there can be little doubt that someone with a fine set of nursing values today will probably perform more effectively later in life than someone with a poor set of nursing values today or that, on the evidence of this piece of work, Nurse Match can be an effective, efficient and systematic way to get at and assess those values.

## CONFLICTS OF INTEREST

The authors report no conflicts of interest. The authors alone are responsible for the content and writing of the article.

## APPENDIX A

### A.1

The outcome of the Nurse Match screening process for the group of respondents is described here. Table A1 lists the group's S^TOT^ scores on each value theme by respondent; respondents are rank ordered on their suitability in terms of their nursing values by their overall (mean) S^TOT^ score.

**Table:** Table A1 All S^TOT^ Scores by Value Theme: Respondents ranked by Mean S^TOT^ Score

Rank	Student	PC	ACC	T	I	CPD	TW	Mean
		S^TOT^	S^TOT^	S^TOT^	S^TOT^	S^TOT^	S^TOT^	S^TOT^
1	SUI0015	98.01	90.39	98.3	93.49	94.09	81.19	92.58
2	SUI0050	93.47	90.98	92.68	87.17	91.96	89.64	90.98
3	SUI0071	87.01	84.45	93.5	80.26	89.86	87.89	87.16
4	SUI0063	86.04	84.33	88.05	86.12	83.1	78.01	84.28
5	SUI0042	83.2	79.59	76.4	84.81	66.43	81.86	78.72
6	SUI0086	91.18	74.72	87.74	62.1	89.48	65.44	78.44
7	SUI0065	82.5	78.09	88.46	75.43	74.72	70.58	78.3
8	SUI0040	79.05	75.81	77.26	76.86	83.41	72.83	77.53
9	SUI0094	84.76	78.47	77.52	70.55	85.27	65.14	76.95
10	SUI0037	90.24	77	70.75	66.94	67.21	79.7	75.31
11	SUI0009	85.09	75.64	68.77	66.43	89.97	65.16	75.18
12	SUI0070	78.94	73.52	76.27	65.83	83.49	69.58	74.61
13	SUI0076	82.42	72.16	84.72	56.88	78.42	70.08	74.11
14	SUI0075	78.38	70.46	76.49	66.98	73.08	68.43	72.3
15	SUI0069	86.44	67.13	60.52	75.38	47.54	83.77	70.13
16	SUI0067	88.55	68.63	61.21	79.42	39.19	76.8	68.97
17	SUI0007	82.32	62.52	65.57	40.21	89.73	68.26	68.1
18	SUI0003	76.81	68.98	70.9	57.47	72.13	58.75	67.51
19	SUI0097	83.88	66.84	69.62	64.68	52.83	66.69	67.42
20	SUI0036	78.01	66.26	75.44	51.89	73.5	55.42	66.75
21	SUI0107	69.98	67.26	61.47	77.58	53.42	69	66.45
22	SUI0054	84.38	64.76	64.85	68.74	42.61	70.13	65.91
23	SUI0073	88.91	68.78	43.57	65.54	47.99	67.56	63.72
24	SUI0023	78.06	63.69	65.26	61.29	52.44	60.81	63.59
25	SUI0091	74.28	60.52	65.19	53.96	66.31	55.1	62.56
26	SUI0103	63.13	59	69.87	56.36	63.73	60.06	62.03
27	SUI0105	70.83	61.84	61.36	52.25	65.89	57.71	61.65
28	SUI0041	73.54	57.64	69.54	57.15	50.04	59.6	61.25
29	SUI0046	94.96	67.36	35	53.72	46.24	69.16	61.08
30	SUI0052	83.42	62.04	59.52	41.38	53.15	66.64	61.03
31	SUI0001	85.61	61.48	50.76	54.48	51.59	60.96	60.81
32	SUI0053	70.43	59.82	51.58	66.25	50.44	66.27	60.8
34	SUI0027	68	66.39	61.79	59.91	55.54	51.01	60.44
33	SUI0021	63.76	58.2	70.38	65.88	49.74	54.57	60.42
35	SUI0079	72.79	57.61	59.59	59.26	49.99	62.65	60.32
36	SUI0028	72.66	58.06	56.27	60.92	50.26	63.36	60.25
37	SUI0059	82.25	59.63	54.64	35.76	67.75	55.67	59.28
38	SUI0078	75.34	56.85	67.15	43.35	56.12	56.46	59.21
39	SUI0035	76.6	59.57	59.4	35.38	67.74	52.16	58.48
40	SUI0072	78.06	56.85	58.4	41.95	57.32	57.14	58.29
41	SUI0095	70.01	59.39	47.46	54.68	57.68	60.21	58.24
42	SUI0055	60.24	57.45	60.27	48.34	59.29	60.56	57.69
43	SUI0012	75.03	56.37	40.02	49.41	60.19	51.46	55.41
44	SUI0087	57.35	52.59	54.66	47.45	61.84	52.29	54.36
45	SUI0014	60.88	51.29	50.07	39.92	58.37	63.79	54.05
46	SUI0029	45.56	54.81	52.09	47.67	75.8	42.74	53.11
47	SUI0033	65.38	48.26	41.51	38.35	54.39	61.78	51.61
48	SUI0077	65.23	52.51	47.32	42.11	46.1	44.58	49.64
49	SUI0056	58.03	53.43	48.07	43.66	37.27	47.04	47.92
50	SUI0017	64.11	44.97	49.29	19.69	61.97	44.62	47.44
51	SUI0032	59.75	44.82	45.74	46.21	37.94	44.79	46.54
52	SUI0064	72.78	36.56	48.32	41.54	23.63	52.04	45.81
53	SUI0068	64.05	45.13	42.05	45.79	28.91	44.69	45.1
54	SUI0085	65.88	46.44	25.6	34.14	31.78	58.48	43.72
55	SUI0006	54.58	43.23	36.03	45.08	35.65	39.85	42.4
56	SUI0018	61.52	40.76	34.38	53.25	9.24	50.75	41.65
57	SUI0047	61.3	43.01	29.32	19.21	45.18	50.16	41.36
63	SUI0096	45.61	39.87	41.83	32.49	41.41	41.5	40.45
58	SUI0026	59.12	33.48	17.16	41.89	15.92	56.62	37.36
59	SUI0005	46.42	35.19	24.25	38.34	43.14	33.7	36.84
60	SUI0093	44.69	34.59	38.44	18.68	50.56	33.07	36.67
61	SUI0058	51.35	33.44	31.94	23.26	42.22	30.31	35.42
62	SUI0004	32.34	13.8	7.07	−12.9	32.71	18.34	15.23
Values mean S^TOT^ score		72.45	59.60	58.07	53.62	57.7	59.6	60.17

Table A2 describes the calculation of the S^TOT^ scores on the six nursing themes using the S‐scores on each value construct.

**Table:** Table A2 Example – Calculation of the S^TOT^ Score by Theme: The Mean S^TOT^ Theme Score is used in overall assessment

Value theme	Respondent SUI0027	S^TOT^	∑ S
PC (8)	S^TOT^ score = (∑S)/80 *100	68.00	54.40
ACC (14)	S^TOT^ score = (∑S)/140 *100	66.39	92.95
T (*6)*	S^TOT^ score = (∑S)/60 *100	61.79	37.07
INT (*5)*	S^TOT^ score = (∑S)/50 *100	59.91	29.96
CPD (4)	S^TOT^ score = (∑S)/40 *100	55.54	22.22
TW *(9)*	S^TOT^ score = (∑S)/90 *100	50.60	45.54
ALL	MEAN S^TOT^	60.37	

## APPENDIX B

### B.2

The findings derived from analysis of the feedback provided in the questionnaire (Figure B1) are summarised in Figure B2. A synopsis of comments in the free text box is provided in section 12.
