# Supplementary material for: Developing nurse match: A selection tool for evoking and scoring an applicant's nursing values and attributes
Source: Nurs Open. 2018 Aug 7;6(1):59–71. doi: 10.1002/nop2.183 (PMC6279733; doi:10.1002/nop2.183)
Supplement: Supplementary file 1 [file NOP2-6-59-s001.docx]

**Supplemental Information**

**Introduction**

To meet word limits and because technical material is more likely to be of value to researchers seeking to replicate the work than to simply interested readers, some of the fundamental and more mathematical information has been gathered in this Supplement.

This amounts firstly, see Table SA, to a list of the bi-polar discourses (value and attribute ‘dimensions’) used to operationalise the value concepts with Table SB showing the preferred pole of each value ‘dimension’, the construct (Cx) that it operationalises and the contribution each value construct makes to the six themed NIEC values.

Secondly the concept of the basic S-score and its calculation is described in some detail including the calculation of the S^TOT^ scores on the NIPEC value themes and the overall score.

**Operationalisation of Nursing Values and Attributes**

Our long-term aim is to bring to nursing a universal, values-based process and instrument that takes account of professional and cultural variance. That is, given localised professional agreement on a set of desirable nursing values, to provide an effective, systematic and reliable way of estimating cultural fit or compatibility of an individual’s attitudes, values and beliefs with the core values and culture of the profession.

The measure to include the level of importance and emotional significance of each professional value to the respondent.

In this pilot one aim was to prove that the concept of the NM S-measure worked well in practice. We researched thoroughly, as reported in the main text, and produced a valid set of values and attributes that was accepted as appropriate by nursing professionals. These were operationalised as twenty bi-polar constructs (value ‘dimensions’) with one pole designated as the professionally preferred pole: see Table SA.

The twenty construct values were reduced using a content analysis to six themed values (see Table SB) deemed appropriate by Northern Ireland Practice and Education Council (NIPEC). Each themed value evolved from the content analysis and presents with a different number of constituent constructs. Only the professionally preferred pole is shown in Table SB but see Table SA for both poles.

The instrument was completed by volunteers from a cohort of first year student nurses. The statistical quality of data and population parameters produced were acceptable.

The purpose of the following section is to describe the NM measure of competence based on responses to these value constructs in some detail: how the estimate of nursing competence was conceived and constructed.

**Table SA: The Bi-polar Attributes and Values Used in the Nurse Match Instrument**

| **Professional Value Contrasting Value** | | |
| --- | --- | --- |
| **C 1** | **Patient dignity and human rights must come first** | **Sometimes medical care needs come before patient dignity and human rights** |
| **C 2** | **The safety of patients must come before anything else** | **Realistically patient safety may suffer because of pressure on staff and hospital resources** |
| **C 3** | **Routine and unpleasant tasks are part of the everyday role of all nurses** | **Routine and unpleasant tasks should normally be the responsibility of less skilled nurses** |
| **C 4** | **Nurses work best when working closely with others in a medical team** | **Nurses work best when their individual competence character and decision making is encouraged** |
| **C 5** | **Can influence people and get them to follow instructions** | **Finds it hard to influence people and get them to follow instructions** |
| **C 6** | **Learning and developing competencies should be a lifelong process for all nurses** | **Learning and developing competencies is mainly for student nurses, qualified nurses mainly deliver nursing care** |
| **C 7** | **Listens carefully and can tune into what others mean** | **Finds listening a distraction and prefers just to get on with the job** |
| **C 8** | **It is better for a nurse to be open and honest in all things** | **Sometimes it is wiser to manage the truth in the greater interest of all concerned** |
| **C 9** | **Resource constraints are no excuse for a lack of kindness compassion and sympathy** | **Accept that pressures can leave less room for kindness compassion and sympathy** |
| **C10** | **One should be prepared to challenge someone more senior if it is felt to be in the interests of the patient** | **One should not challenge someone more senior in any circumstances** |
| **C11** | **Enjoys making decisions within their area of competence** | **in a shared area of competence sometimes prefers the other person to take decisions** |
| **C12** | **Owns their work and takes personal responsibility for their judgement and action** | **Adheres strictly to guidelines and instructions which are at fault if things go wrong** |
| **C13** | **One should take the time needed to do the task properly** | **One must be quick and do the best one can with the task in the time available** |
| **C14** | **Good communicator, always understood** | **Not a good communicator, often misunderstood** |
| **C15** | **Relates well to others** | **Often misunderstands and has problems relating to others** |
| **C16** | **Can be relied upon** | **Real world can affect reliability** |
| **C17** | **Does the job with minimal supervision** | **Works best when managed by others** |
| **C18** | **Generally, understands situations** | **Sometimes misunderstands situations** |
| **C19** | **Often pauses and reflects on how things have gone** | **Rarely takes the time to reflect on how things have gone** |
| **C20** | **Is always thinking about the other person** | **Focuses on their own needs and priorities** |

**Table SB: Value Themes: Scoring Matrix Showing Contribution of Bi-polar Constructs (C1 … C20) to value themes**

| **Value theme/** | **Person Centred** | **Accountable** | **Trust** | **Integrity** | **Committed** | **Team** | **Total** | **Professional nursing value or attribute used as one pole of Construct** |
| --- | --- | --- | --- | --- | --- | --- | --- | --- |
|  |  |  |  |  | **to personal** | **Work** | **Use of C** |  |
| **Construct** |  |  |  |  | **development** |  |  |  |
| **C1** | **X** | **X** | **X** |  |  |  | **3** | **Patient dignity and human rights come first** |
| **C2** | **X** | **X** | **X** |  |  |  | **3** | **Patient safety comes before anything else** |
| **C3** |  | **X** |  |  |  | **X** | **2** | **Routine/unpleasant tasks part of role of all nurses** |
| **C4** |  |  |  |  |  | **X** | **1** | **Nurses work best as a medical team** |
| **C5** | **X** | **X** |  |  |  |  | **2** | **Can influence people and get them to follow instructions** |
| **C6** |  |  |  |  | **X** |  | **1** | **Learning and developing competence is a lifelong process** |
| **C7** | **X** |  |  |  |  |  | **1** | **Can listen carefully and can tune in to what people mean** |
| **C8** |  | **X** |  | **X** |  |  | **2** | **Are always honest** |
| **C9** | **X** | **X** |  |  |  |  | **2** | **Are kind compassionate sympathet6ic; resources no excuse** |
| **C10** |  | **X** |  | **X** |  |  | **2** | **Are prepared to challenge senior in interest of patient** |
| **C11** |  | **X** |  |  | **X** |  | **2** | **Enjoy making decisions within their area of competence** |
| **C12** |  | **X** | **X** |  | **X** |  | **3** | **Take personal responsibility for judgement and action** |
| **C13** |  | **X** | **X** | **X** |  |  | **3** | **Take time necessary to do a job properly** |
| **C14** | **X** |  |  |  |  | **X** | **2** | **Are good communicators, always understood** |
| **C15** | **X** |  |  |  |  | **X** | **2** | **Relate well to others** |
| **C16** |  | **X** | **X** | **X** |  | **X** | **4** | **Can be relied upon** |
| **C17** |  | **X** | **X** | **X** |  | **X** | **4** | **Do work with minimum supervision** |
| **C18** |  | **X** |  |  |  | **X** | **2** | **Generally, understand situations** |
| **C19** |  | **X** |  |  | **X** | **X** | **3** | **Often pause and reflect on how things have gone** |
| **C20** | **X** |  |  |  |  | **X** | **2** | **Are always thinking about the other person** |
| **C by theme** | **8** | **14** | **6** | **5** | **4** | **9** |  |  |

**The Concept of the S-score**

Each of the six value themes is a composite of several NM values and is aligned with NIPEC attributes, (see Table SB above for the full breakdown - the number of NM elements is indicated there e.g. 8 for Person Centred (PC) theme). NM values are presented as a choice between two alternative interpretations of the nursing value, one of which is welcome the other less so – see the more comprehensive explanation below*.*

The measure was designed to estimate the significance (S) of each NM nursing construct to the respondent i.e. significance is a product of the stability of meaning of the attribute (sp.) and its emotional significance (es.) to the respondent. The sign of the product being determined by the suitability of the preferred response. So, for a bi-polar construct C, S = + (C**_sp._** * C**_es._**) with the positive sign being determined by a choice of pole designated as professionally appropriate. Choosing the less welcome pole results in S = - (C**_sp_**. * C**_es_**.).

**The calculation of the S-scores**

The estimate of the significance (S**^TOT^**) of a themed nursing value being simply the sum of the set of S-scores (**∑S**) on its constituent attributes: see Table A2. To standardise theme scores the S**^TOT^** score is presented as a percentage of the maximum possible S-score for a theme e.g. for SUI0027 the sum of S-scores on Team Work (TW) was 45.54. Theme TW has nine (9) attributes (maximum score on each is 10) = 90. S**^TOT^** = **∑S**/90*100 = 45.54/90*100 = 50.60.

**Table SC: Calculation of a S^TOT^ score estimating suitability for nursing by value theme.**

| **VALUE THEME** | **Respondent SUI0027** | **S^TOT^** | **∑ S** |
| --- | --- | --- | --- |
| **PC (8)** | **S^TOT^ score = (∑S)/80 *100** | **68.00** | **54.40** |
| **ACC (14)** | **S^TOT^ score = (∑S)/140 *100** | **66.39** | **92.95** |
| **T (*6)*** | **S^TOT^ score = (∑S)/60 *100** | **61.79** | **37.07** |
| **INT (*5)*** | **S^TOT^ score = (∑S)/50 *100** | **59.91** | **29.96** |
| **CPD (4)** | **S^TOT^ score = (∑S)/40 *100** | **55.54** | **22.22** |
| **TW *(9)*** | **S^TOT^ score = (∑S)/90 *100** | **50.60** | **45.54** |
| **ALL THEMES** | **MEAN S^TOT^** | **60.37** |  |

**A more comprehensive explication of the Nurse Match S-score measure**

NM measures the significance (S) to a respondent of nursing values and attributes such as compassion, truthfulness and reliability by pointing up the personal meaning of truthfulness, say, and strength of feeling associated with it.

To do so NM combines two measures from the theory of ISA; (a) stability of meaning and (b) strength of feeling associated with value ‘dimensions’ such as contrasting attitudes to honesty, C8 in Table SA.

More precisely, to achieve this NM draws on three concepts from Identity Structure Analysis (ISA): see Weinreich, P., & Saunderson, W. (Eds.). (2005)

• ‘Structural Pressure’ (sp.) a measure of the importance of a value to the respondent in appraising others and making sense of life experience (its usefulness and so stability)

• ‘Emotional Significance’ (es.) a measure of the emotional significance of a value to the respondent and

• The ‘Preferred Pole’ (PP) of the value; the personally preferred alternative on the bi-polar value dimension.

Twenty nursing values are clustered to form six value themes; person centredness, accountability, trust, integrity, commitment to personal development and teamwork: see Table SB above.

Each value is presented as two contrasting attitudes on a zero-centred semantic differential scale presenting the value ‘dimension’ as a bi-polar construct. All entities are appraised by the respondent using the value dimensions, resulting in a 20 x 13 matrix of scores. The algorithms defining ‘structural pressure’ and ‘emotional significance’ draw on this data to calculate a score for sp. and es. on each construct.

Similarly, an algorithm based on a respondent’s appraisal of ‘aspirational self’ is used to determine the personally preferred pole on a value dimension.

If the respondent’s ideal personal preference (V**_IPP_**) is on the professionally preferred pole (V**_PROP_**) that attracts a positive sign to S (+S) indicating a welcome attribute. An ideal personal preference (V**_IPP_**) on the contrasting pole (V**_OP_**) is regarded as unwelcome and attracts a negative sign to S (-S). Thus, the polarity of the chosen response on a value will determine whether the S-score on that value will increase or reduce the overall score (S**^TOT^**) on a value theme.

Accordingly, where V is a nursing value, V**_sp_** is the structural pressure on a value and V**_es_** the emotional significance of a value, the S-score for V is calculated as follows:

when the ideal personal preference (V**_IPP_**) is a professional value (V**_PROP_**):

S = + (V**_sp_** x V**_es_**)/100 and

when the ideal personal preference (V**_IPP_**) is the contrasting pole (V**_OP_**) i.e. not the preferred professional value the S-score is negative:

S = - (V**_sp_** x V**_es_**)/100.

The respondent’s S-score on a value theme with n constituent values S**^TOT^** = $\sum_{i=1}^{n} S.$

S-scores for value themes are converted into a percentage of the maximum possible S**^TOT^** score for practical reasons: see Table A2 above for an example of a set of results.

The sp. score ranges from + 100 to – 100. The es. score ranges from 0 to 10. S**^MAX^** (maximum score on a value) is set as 10 for practical purposes ((100 x 10)/100) = 10.

S**^MIN^** (the minimum score) would therefore be – 10. However, the lower is the sp. score on a value the more conflicted and uncertain is its use by the respondent. Consequently, S**^MIN^** is constrained by a cut off at zero. An sp. score of zero or less (sp. <0.01) indicates too much uncertainty about the importance of the value to the respondent so that an S-score of zero is regarded as the fairest outcome (e.g. sp.* es. = -20.3 * es. = 0 * es. = 0).

The calculation of S**^TOT^** ((($\sum_{i=1}^{n} S )/\left( n*10 \right))*100) is straightforward.$ For example, in respect of the themed nursing value ‘Commitment to personal development’ (CPD) there are four constituent professional values: C6 ‘developing competencies is a lifelong process; C11 ‘enjoy making decisions and showing competencies’; C12 ‘takes personal responsibility for their actions’; C19 ‘often pauses to reflect on how things have gone’. So, for CPD: S**^TOT^** = (S**^C6^** + S**^C11^** + S**^C12^** + S**^C19^**)/40*100 where S is the S-score on a construct (C).

The S**^TOT^** scores range from +100 to -100: a complete match and total engagement with all constituent professional values to a complete mismatch. See Figure 2 main text for cohort S**^TOT^** means on value themes. Overall mean score on value themes for this cohort was S**^TOT^** = 58.89.

The mean of the set of S**^TOT^** scores for themed values is a single figure estimate of the quality of an applicant’s personal belief in and emotional commitment to nursing values and attributes. Further, the set of S**^TOT^** scores on value themes provides a profile of the respondent’s nursing attributes. And the S-scores on a theme’s constituent values offer a deeper level of insight if it is required.

Beyond that, an individual’s overall score and scores on themes can be compared with the cohort or group scores to get a sense of where norms are and where differences from the norm lie.

Eventually it may be possible to create a psychometric test based on this instrument, measuring nursing attributes standardised on a wider population but in the interim and probably wisely the cohort of annual applicants locally (or subsequently a group of nurses of research interest) are the populations whose parameters we should be interested in.
